# Underwater noise emissions from ships during 2014–2020^☆^

**DOI:** 10.1016/j.envpol.2022.119766

**Published:** 2022-10-15

**Authors:** Jukka-Pekka Jalkanen, Lasse Johansson, Mathias H. Andersson, Elisa Majamäki, Peter Sigray

**Affiliations:** a Atmospheric Composition Research, Finnish Meteorological Institute, P.O. Box 503, FI-00110 Helsinki, Finland; b Underwater Technology, Division of Defence Technology, Swedish Defense Research Agency, Stockholm, Sweden; c Marine Robotics Laboratory, Engineering Mechanics, Royal Institute of Technology, Stockholm, Sweden

**Keywords:** Shipping, Underwater noise, Noise energy emissions, Noise sources, Source modeling

## Abstract

This paper reports trends in the input of underwater noise source energy emission from global shipping, based on bottom-up modeling of individual ships. In terms of energy, we predict the doubling of global shipping noise emissions every 11.5 years, on average, but there are large regional differences. Shipping noise emissions increase rapidly in Arctic areas and the Norwegian Sea. The largest contributors are the containerships, dry bulk and liquid tanker vessels which emit 75% of the underwater shipping noise source energy. The COVID-19 pandemic changed vessel traffic patterns and our modeling indicates a reduction of −6% in global shipping noise source energy in the 63 Hz ⅓ octave band. This reduction was largest in the Greenland Sea, the Coastal Waters of Southeast Alaska and British Columbia as well as the Gulf of California, temporarily disrupting the increasing pre-pandemic noise emission trend. However, in some sea areas, such as the Indian Ocean, Yellow Sea and Eastern China Sea the emitted noise source energy was only slightly reduced. In global scale, COVID-19 pandemic reduced the underwater shipping noise emissions close to 2017 levels, but it is expected that the increasing trend of underwater noise emissions will continue when the global economy recovers.

## Highlights

• Global underwater noise emissions from shipping doubled in just 11.5 years.
• Underwater noise emissions from ships increase rapidly in Arctic areas.
• A large variability exists in shipping noise emission trends of different regions.
• COVID-19 pandemic decreased shipping noise emissions back to 2017 levels globally.
• Containerships are the largest contributor to shipping noise emissions.

## Graphical abstract

**Figure:**
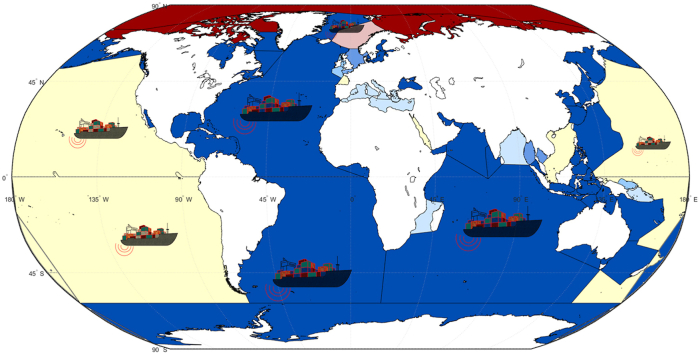

## 1 Introduction

The disruption of shipping after the notorious September 11^th^ attack on the World Trade Center in 2001 was labeled as “An irreproducible experiment” considering how marine life reacted to an unexpected silent period in North American sea regions (Rolland et al., 2012). In 2020, global COVID-19 pandemic brought with it a global disruption, which changed the traffic patterns of shipping and ground almost the whole cruise sector to a halt. It is expected that this global disruption of movement of goods and passengers have a widespread effect in the shipping sector. Regional lockdown periods and travel restrictions reflect strongly on ship movements, especially those concentrated on passenger traffic. While the Sep 11^th^ 2001 events led to marine traffic restrictions mostly concentrated in North American coastal regions, the ongoing COVID-19 has global consequences. It is not currently known how marine life reacted to this unexpected change in shipping intensity of 2020, but it is widely recognized that noise impact on marine life ranges from masking of communication, stress to behavioral changes and may ultimately lead to adverse effects on population level (Duarte et al., 2021).

Many of the subsectors of shipping respond differently to disruptions such as COVID-19. Further, the timing of regional lockdowns inevitably reflects on the shipping patterns, but these may occur in different seasons which implies the spread of the pandemic throughout the various parts of the world. In general, the United Nations Conference for Trade and Development (UNCTAD, 2020) recently projected a large trade contraction for 2020, which eclipses even the economic crisis of 2008. Regardless of the overall view of the maritime trade and some of its subsectors (Notteboom et al., 2021), a comprehensive view on shipping noise is missing. Since various shipping sectors have non-uniform response to the pandemic, also the contributions to shipping noise will differ. In addition to these unexpected large-scale changes of ship traffic, voluntary regional examples of ship traffic changes affecting underwater noise exist. These include e.g. the Balinese “Day of silence”, International Quiet Ocean Experiment (Boyd et al., 2011) and the Canadian experiment within the Southern Resident Killer Whale Critical Habitat (Matthews et al., 2018).

The Automatic Identification System (AIS) has been used in various noise studies (Gaggero et al., 2015; Garrett et al., 2016; Jalkanen et al., 2018; Leaper, 2019; McKenna et al., 2012; Mustonen et al., 2019; Prins et al., 2016; Veirs et al., 2016; Williams et al., 2021), but to our knowledge mostly to identify vessels and compute distances to hydrophones and not for global reporting of shipping noise. Currently available modeling tools may help to extend the noise reporting to the global domain but require validation measurements (Erbe et al., 2014; Karasalo et al., 2017; Macgillivray and de Jong, 2021; Williams et al., 2015, 2014). A detailed breakdown of shipping contribution to greenhouse gas emissions (GHG) has been regularly done for atmospheric pollutants (Faber et al., 2020), but similar level of detail is also available for noise emission modeling. The source modeling efforts may provide insight on the environmental pressures, such as shipping noise, but this is not enough to conduct a comprehensive impact analysis.

A commonly used method in ecology is to assess the environmental impact based on a pollutant that spreads into the environment affecting part of the species habitat. This method consists of several steps where the first is to characterize the properties of the pollutant source; both the source strength and the spatial and temporal scales must be estimated. The second step is to determine the pressure at the receiver. In the last step impacts on habitat or the population are estimated (Duarte et al., 2021). In this paper only the first step is estimated. It can be used as guidance to point out areas of high shipping noise, but detailed studies of impacts should be based on observations of environmental state.

For underwater noise, attempts have been made to describe the anthropogenic contributions (Hildebrand, 2009; Miksis-Olds and Nichols, 2016), especially shipping (Audoly et al., 2017; Erbe et al., 2014; Gervaise et al., 2015; Hatch et al., 2008; Leaper, 2019; McKenna et al., 2012; Porter and Henderson, 2013, 2014; Sertlek et al., 2019; Williams et al., 2014, 2015), but these are rarely available at global level even when using a proxy variable like Gross Domestic Product (GDP) (Frisk, 2012). One of the first regional attempts to systematically map a regional soundscape was made in the BIAS project for the Baltic Sea (Mustonen et al., 2019). During the full year of 2014, continuous measurements of sound levels were performed at 38 locations in the Baltic Sea. These measurements were used to calibrate an acoustic model that produced monthly statistics in the form of soundscape maps based on AIS and Vessel Monitoring System (VMS) data. The study was limited to soundscape maps for the 1/3 octave band center frequencies 63, 125 and 2000 kHz. Farcas et al. (2020) studied the excess levels related to masking in the North Sea and produced total noise and ship noise excess maps based on AIS data. Their approach followed the BIAS methodology but expanded the investigating with a thorough frequency analysis. The study was however, limited to the coastal area were AIS coverage was assumed to be satisfying. Pennino et al. (2017) combined habitat modeling and ship traffic to assess the impact on the bottlenose dolphin, stripped dolphin and fin whale, in the Bonifacio Strait by investigating the overlap between mammal habitat and spatial distribution of ships. This study did not make use of either noise propagation modeling or an impact assessment but identified hot-spot areas where overlaps were large.

Considering the increasing trend of ship traffic, it is unlikely that shipping noise would decrease unless incentives or regulatory steps are introduced. Underwater noise emissions from ships are currently not regulated, but they are recognized as an arising environmental problem (IMO, 2014; Matthews et al., 2018). The necessary background studies for policy changes are lacking. For example, the awareness of global underwater noise emissions from shipping in recent years is largely missing, which makes it difficult to assess the costs and benefits of potential changes to current policies. Long-term observations of shipping noise covering large sea regions are only starting to emerge, even if wide scale monitoring has been done routinely for military purposes. Vessel noise decreases with vessel speed, which has been suggested as one of the methods to reduce vessel fuel consumption and emissions (Leaper, 2019; Leaper et al., 2014; MacGillivray et al., 2019). This may not apply to all ship types, because not all ships adjust their speed by altering propeller rotation speed (Traverso et al., 2017).

The aims of this paper include: **First**, provide a view to current underwater noise source energy emissions from ships, together with the impacts of COVID-19 pandemic to vessel noise emissions. **Second**, to generate datasets for spatial distribution of shipping noise emissions and its long-term trend by sea region. **Third**, analyze traffic pattern changes and ship type contributions to underwater noise emissions. It should be stressed that this paper is solely focused on the spatial and temporal characteristics of ship noise emissions.

As a continuation, the approach presented in this paper can be applied routinely for any marine location with AIS data coverage, thereby enabling further research of impacts on marine life, such as effects of chronic exposure, behavioral response, communication, and habitat degradation.

## 2 Materials and methods

The Ship Traffic Emission Abatement Model (STEAM) of Finnish Meteorological Institute (FMI) was used in this work (Jalkanen et al., 2009, 2012; 2018; Johansson et al., 2013, 2017). Input data for the model, the vessel activity and fleet description, were obtained from Automatic Identification System (AIS) data provided by Orbcomm Ltd. and IHS Markit, respectively. The STEAM model predicts instantaneous vessel power use, based on ship identity, vessel description and speed indicated by AIS position reports. The model describes the overall state of the vessels and their engines considering relevant environmental regulations. Previously, this approach has been used to estimate emissions to air, discharges to the sea and underwater noise emissions.

The Orbcomm AIS dataset used for vessel activity description consisted of 3.1 billion AIS position reports each year (average of message counts each year during the period 2014–2020) and includes data from both terrestrial and satellite AIS receivers. The use of AIS equipment is compulsory for large ships, but optional for small vessels or those operating on national waters. The global dataset used in this study includes AIS reporting of large IMO registered ships as well as those of small vessels, but not all waterborne traffic is required to use an AIS transponder. The description of noise from small vessels is likely to be underestimated in our approach.

STEAM estimates vessel noise source levels using the Wittekind noise source model (Jalkanen et al., 2018; Wittekind, 2014) which describes low- and high frequency cavitation and machinery contributions separately. In the Wittekind model, vessel speed affects the noise source levels (in dB re 1 μPa) and the model predicts significant increase if cavitation inception speed (CIS) is exceeded. The Wittekind model requires determination of vibrating engine mass, engine-mounting type, number of operating engines, vessel displacement and most importantly, the cavitation inception speed as input. The use of commercially available databases of ship technical descriptions offers a more complete description of vessel features, physical dimensions, propulsion and machinery details than what is available in AIS data itself (Gaggero et al., 2015; Macgillivray and de Jong, 2021). Most of the required parameters for the Wittekind noise source model are readily available for the model. A short summary of required additions is provided in the Supplement, but our earlier works (Jalkanen et al., 2018; Karasalo et al., 2017) contain additional details of the methodology and comparison to hydrophone measurements. Throughout this paper, the noise source energy emitted by a ship to the sea is considered as noise emissions and are reported in energy units (Joules) because the duration of the noise signal is also considered. The key benefits of the used modeling approach include: **a)** the use of transponder data from AIS, which describes the ship activity as a function of time; **b)** updates of global underwater noise emission inventories, which can be reported annually; **c)** realistic description of noise as a function of vessel physical and technical description and **d)** construction of noise scenarios, which allow testing of vessel based mitigation options.

The challenges of the chosen approach include an estimation of CIS and engine mounting parameters needed by the Wittekind noise source model, which cannot be obtained from available vessel databases. The approach used in this paper excludes the noise shipping generates during icebreaking, which can be significant, but it is mostly restricted to polar areas and dwarfed by the continuous shipping noise. It should also be stressed that noise source energy maps presented in this paper do not include noise propagation but are time integrations of noise as energy on a sphere around the source. The source level is defined at 1 m distance the energy is calculated by integration of the source level on a sphere using the source level. Both the source level and the energy are thus hypothetically placed in an infinite uniform lossless ocean, following the definition in ISO18405 (2017). When instantaneous noise is integrated over time, a noise source energy map is obtained (Jalkanen et al., 2018) which can be used to understand the geospatial distribution of vessel noise source energy. This is a cumulative noise source energy assessment with an integrating period of one year (total noise source energy) or one day. The work reported in this paper involves description of noise sources and their time integration as an anthropogenic environmental pressure, which can be used as a basis for further work but should not be taken as a description of environmental state.

In STEAM, there exists an option to generate output of shipping noise as point sources, but this feature was not used in the current work and gridded outputs were produced instead, because noise propagation studies were not conducted. The current dataset is for 2014–2020, but regular annual updates are possible in scales from local to global.

## 3 Results and discussion

### 3.1 Geographical distribution of global shipping noise emissions

This work is based on the global modeling of noise source energy output of individual ships. In the results, the noise source energy is aggregated to daily grids with a resolution of 0.1° (WGS84 coordinate system). The noise emissions were calculated as Gigajoules (1E9) of total emitted acoustic energy (in gigajoules) over a year and a specified area, in a specified frequency band (Jalkanen et al., 2018). These gridded data were produced for 63, 125 and 2000 Hz center frequencies of ⅓ octave bands and the data generated are available for further study. All modeling was done at vessel level, which enabled studies of noise emissions by vessel type, age, flag state or size. In consecutive sections, the overall geographical distribution of shipping noise emissions, its temporal variation and changes caused by the COVID-19 pandemic are presented.

**Fig. 1:**
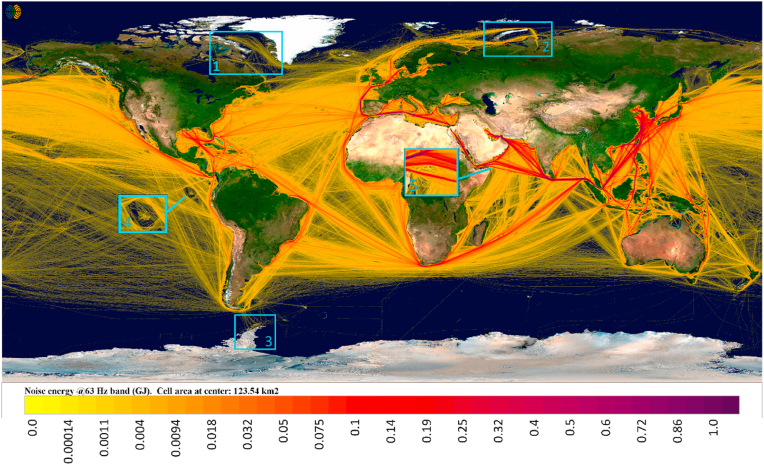
**Global map of underwater noise emissions from ships in 2019 (63 Hz 1/3 octave band, in Gigajoules).** The labeled areas are 1: Baffin Sea with Milne mining operations; 2: Kara Sea with Yamal gas fields; 3: Palmer basin research stations; 4: Galapagos Islands; 5: Socotra Island.

In Fig. 1, the geographical distribution of global underwater noise source energy emissions from ships (63 Hz 1/3 octave band) to the sea is presented. The main shipping lanes, e.g. the ones from China via the Malacca Strait and Red Sea to Europe, have the highest noise contributions from shipping. Other noisy areas are the Gulf of Mexico and the shipping lanes from Malacca Strait towards Madagascar and South Africa. In the Arctic, both the Barents and Kara seas have significant noise contributions from ships, most likely connected to oil and gas extraction at high latitudes, and to less extent, usage of the northern sea route. In addition, the noise source energy emissions at Baffin Bay, likely connected to the increased Milne mining operations can be seen in Fig. 1. Very few ships attempted sailing the northwest passage (Halliday et al., 2017). Shipping noise in the Antarctic area is connected mostly to the service traffic of various research stations near Palmer Basin. There are very few places unaffected by shipping noise; even in the protected area of Galapagos Islands, there are indications of shipping noise patterns which connect the individual islands.

**Fig. 2:**
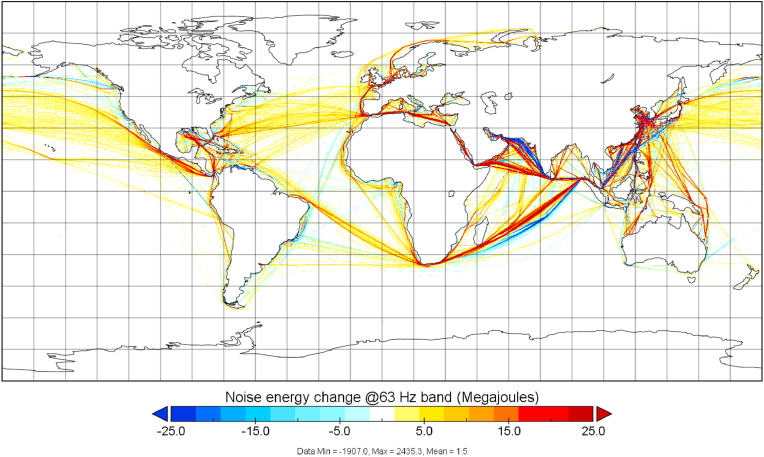
**Changes in underwater noise source energy emissions, 2014–2019, at 63Hz 1/3 octave band (in Megajoules)**. This difference map illustrates the changes during this period. Red areas indicate increase in shipping noise and blue areas signal a decrease.

In Fig. 2, the difference of annual total noise source energy from ships between years 2014 and 2019 is shown. This has been done simply by subtracting the annual totals of 2019 from the totals of 2014 (63Hz 1/3 octave band data). Therefore, negative values indicate a reduction of noise source energy and positive values an increase, respectively. It can be seen from Fig. 2 that in most areas the annual shipping noise emissions have increased. However, there exist few places where emissions have reduced during the study period, such as parts of the Gulf of Oman, but this is likely a result of increasing political tension in the area than an attempt to reduce noise. The main shipping lane in that area was further south in 2019 than in 2014. Also, the noisiest areas in the shipping lane from Malacca Strait towards the southern tip of Madagascar has shifted closer to the islands of Reunion and Mauritius between 2014 and 2019, which has increased the shipping contributions in areas close to these two locations. Further, significant increase in underwater noise emissions was observed from Asia-Europe traffic between the Horn of Africa and Socotra island.

An increase in emitted noise was predicted for the South China Sea, Yellow Sea, and the Mediterranean Sea. Despite some regional differences in underwater noise emission patterns, increased emissions were discovered in most sea areas from 2014 to 2019. At global level, the underwater noise emitted by ships has doubled in the period of 11.5 years for this frequency band, which is in line with the often quoted observations of +3 dB/decade rate, and corresponding to doubling of energy, for the Northeast Pacific (McDonald et al., 2006). The observed +3 dB/decade rate is not directly associated with noise emissions, but our results indicate non-uniform regional development of noise emissions. Eastern and Southeastern Asia regions have large underwater noise emissions, especially Singapore and Hong Kong-Shanghai shipping lanes indicate high contribution of ships to underwater noise.

### 3.2 Temporal distribution of global shipping noise emissions

**Fig. 3:**
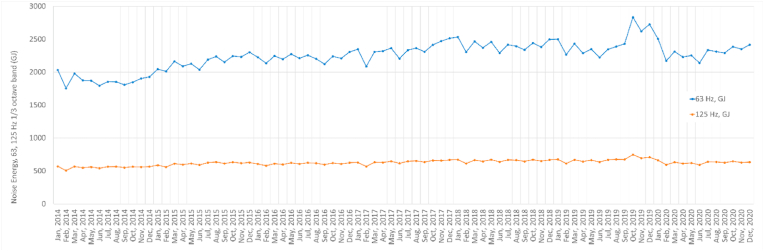
**Monthly emissions of global underwater noise at frequencies 63 and 125 Hz of 1/3 octave bands.** The global COVID-19 pandemic decreased the noise emissions significantly from Oct–Nov 2019 onwards.

As shown previously (Jalkanen et al., 2014), there are seasonal patterns in regional ship exhaust emissions, but similar features are also observed for noise. The temporal profile of cargo traffic is different from that of passenger traffic, and these features are prominent in areas with dense passenger shipping. For example, in the Baltic Sea area, the summer season represents the maximum when passenger cruise traffic is at its highest and air emissions from ships are high. With noise emissions, the temporal variation can be as high as 19% at monthly level, using daily corrected values. Fig. 3 indicates the seasonal development of noise at global level and reports monthly totals for shipping noise emissions. It can be observed that the highest monthly emissions (for the 63Hz frequency at 1/3 octave band) mostly occur in Oct–Nov each year, but the overall trend throughout the whole 2014–2019 period is increasing. If the increasing trend is continued, global shipping noise source energy emissions will double in a period of 11.5 years.

### 3.3 Impact of COVID-19 on shipping noise emissions

The increasing trend observed for the 2014–2019 period for the global domain was broken by the COVID-19 pandemic. This caused a disruption in shipping activities, which, in turn, resulted to a decrease in reduced the noise emissions from ships nearly to 2017 levels. Recently, studies reporting decreased shipping noise in various areas have appeared (Čurović et al., 2021; Thomson and Barclay, 2020) which could be used to understand changes the pandemic introduced to underwater noise in different areas. These studies were conducted as hydrophone measurements for the first quarter of 2020 indicated a reduction in vessel noise, which was attributed to the traffic reduction. It is expected that this decrease of underwater noise is only temporary and upon the recovery of the world economy, noise emissions will be increased again. This is probable unless vessel operation and fleet size changes as a response to greenhouse gas (GHG) reduction efforts.

**Fig. 4:**
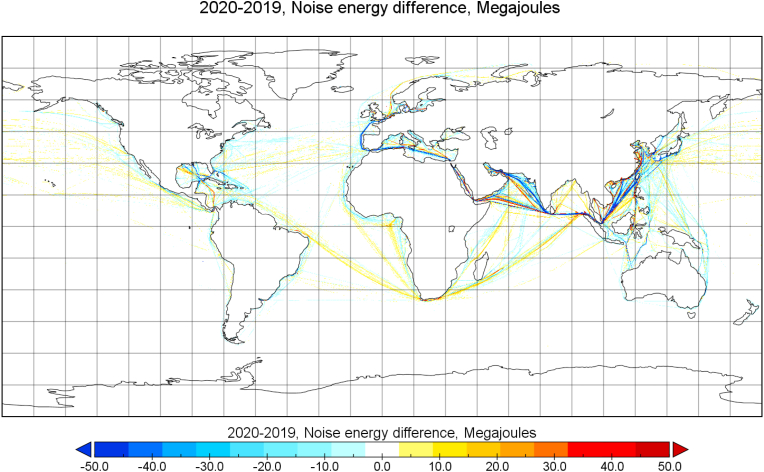
**Changes in underwater noise source energy (63 Hz 1/3 octave band) emitted from ships in 2020**–**2019 (in Megajoules).** This image is a difference plot of annual noise source energy emitted in 2020 and 2019. Noise source energy emissions are given in units of megajoules per grid cell. The COVID-19 pandemic decreased the underwater noise emissions significantly in major shipping routes.

The reduction of underwater noise emissions from ships because of the global pandemic began in November 2019 (Fig. 3). Global shipping noise source energy reached its maximum in October 2019 and started to decrease thereafter, with a decrease in Dec 2019–Feb 2020. This disrupted the increasing trend of underwater noise emissions and the total noise emissions were returned close to the level predicted for 2017. This disruption was experienced at different times, depending on the extent and the timing of regional lockdowns. In Fig. 4, the reduction of underwater noise emissions from ships (at 63Hz frequency of ⅓ octave band) is visible on major shipping lanes between China and the EU (Arabian Sea: −8%, Red Sea: −5%, Mediterranean Sea: −9%).

The emissions of shipping noise on Eastern China Sea (−3%) were only slightly changed and in some sub-regions, like the Gulf of Thailand, noise emissions increased (+13%) despite the pandemic.

A separate analysis for the EU, Mediterranean, North Sea and the Baltic Sea was conducted based on the global noise and CO_2_ emissions. Overall, the underwater noise emissions from ships at EU region was reduced by −8% starting from March 2020 (63 Hz 1/3 octave band). In December 2020, the monthly noise source energy emission levels in the EU domain were reduced by −6% compared to the noise emissions in January 2020. Closer inspection of regional seas like the Baltic, North Sea and the Mediterranean Sea indicate the largest noise emission reduction occurred in the Baltic Sea area (−14%), followed by the Mediterranean Sea (−9%) and the North Sea (−5%). The largest interannual decreases in the noise emissions from shipping in these areas were observed during the summer months. The noise emissions from the Baltic Sea and the Mediterranean Sea shipping had a temporary increase in autumn 2020, after the June–July 2020 minimum, but decreased again towards the end of 2020. In the North Sea, a decreasing trend was predicted throughout the year 2020. The decrease in noise emissions towards the end of 2020 coincides with the start of the second wave of lockdowns in Europe (Looi, 2020).

### 3.4 Regional trends of underwater shipping noise emissions

**Fig. 5:**
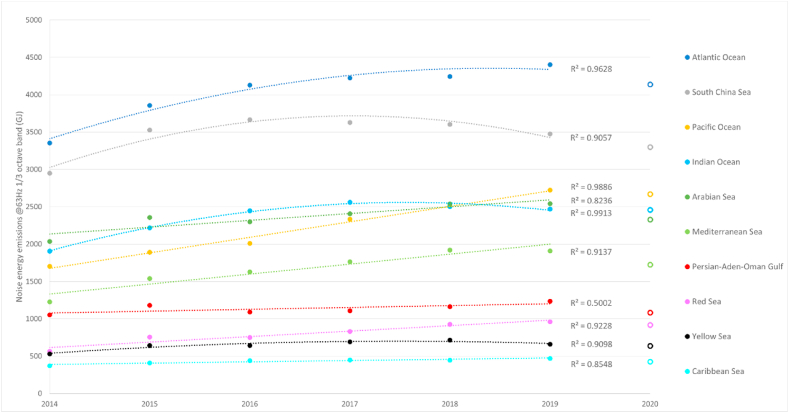
**Regional trends of underwater noise source energy emitted by ships in 63Hz frequency at 1/3 octave band.** Increasing noise emissions are observed in most sea areas. Open symbols are noise energies for same sea regions in 2020, but these have not been included in the estimation of the trend. Note, that a parabolic function leads to a better fit than a linear trendline in some cases.

Previous measurements in Northeast Pacific over four decades indicated an increasing 3 dB/decade trend (doubling of noise every ten years), which has been viewed as moderate growth of shipping noise (McDonald et al., 2006). For comparison, we have computed the annual noise source energy emitted from ships in selected sea regions – including the Pacific - during 2014–2019 (Fig. 5) using the sea area definitions from the International Hydrographic Organization (IHO). The long-term development of noise is different in various areas and a single number, like the 3dB/decade, does not describe the heterogeneous trends very well. Table S3 of the Supplementary data contains the regional data in numerical form.

The increase of regional ship underwater noise emissions in the 2014–2019 period was found to be diverse in various parts of the world. Based on the global modeling of ship underwater noise emissions, the global trend from 2014 to 2019 indicates that noise emissions may double every 11.5 years, but regional variations of noise increase are large (Fig. 5). The global pandemic disrupted the rapidly increasing noise trend and returned the noise emissions close to 2017 level.

At the global level, the shipping noise emissions increased at a rate which is agreement with the trend reported previously, but regional imbalance of noise emissions is significant. The regional noise emission energy totals by sea area were investigated and are depicted in Fig. 5. The closed symbols correspond to annual noise emissions (63Hz 1/3 octave band) from ships in different IHO sea areas during 2014–2019. The symbols for year 2020 data are coloured similarly to those of earlier years. The trend lines, based on regression totals, include data from the period before the pandemic and could be considered to reflect a period with regular shipping without major disruptions to vessel traffic. According to the results, there has been an increase in vessel noise emissions in many sea areas and for most of the presented sea areas the trend is linear. If these trends were to continue, without the COVID-19 impact on noise, it would take about one decade to double (+3 dB) the noise emissions, but various sea regions may experience different periods of noise source energy doubling. This analysis can easily be updated when global AIS data becomes available for 2021 and beyond, to confirm whether the reduction of noise emissions is temporary or permanent.

Based on the trends shown in Fig. 5 it is possible to estimate the time which it takes to double the underwater noise source energy emissions (see Table S1 for numerical values). Based on this analysis, three different groups of sea regions can be observed. First there is the group (29 areas) where the trendline of shipping noise emissions indicates an either a decreasing trend during the study period or the slope of the trendline indicates doubling of shipping noise emissions during a period of two decades or longer. In this group, the Bering Sea, sea areas around Alaska and the Sea of Azov (north of the Black Sea) and Gulf of California have decreasing noise emissions, and the time period for noise emission doubling is rather long for Indian Ocean (21 years), Yellow Sea (30 years) and Gulf of Mexico (39 years). The second group consists of sea regions, where the slope of the trendline leads to doubling of noise source energy emissions every 10–20 years. This group consists of nine regions, including many areas in Europe and Asia. The third group contains six sea areas investigated in this study and the doubling of vessel noise emissions takes less than a decade. This group contains the Pacific Ocean, the Red Sea and the Arctic Sea. Especially, in the Arctic areas the annual noise source energy growth is significantly faster than the 3 dB/decade rate. This group includes not only the Arctic, but also the Norwegian Sea. The noise source energy totals in the Arctic Sea region in 2014 were low and doubling of shipping noise emissions can be achieved rather easily. Regardless, it should be noted that most of this increase in northern latitudes is probably a result of increased traffic towards the Barents and Kara Sea and are consequences of increased exploitation of natural resources in that area.

**Fig. 6:**
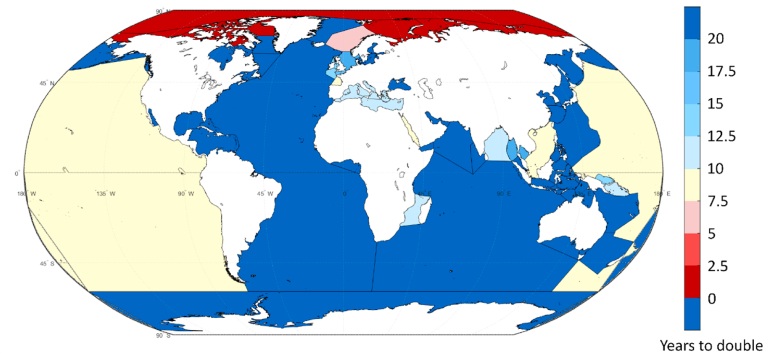
**Underwater noise emissions trend in various sea areas.** Shades of blue = Doubling of shipping noise emissions takes more than 10 years, or it has decreased over time; Light yellow = noise emissions double within 7.5–10 years; Light red = noise emission doubling within 5–7.5 years; Dark red = noise emissions doubling in less than 3 years; It should be noted that Arctic shipping noise emissions in 2014 started at a very low level and modest increase in Arctic shipping easily doubled the noise emissions from ships.

The regional differences in noise emission doubling are evident from Fig. 6. The high latitudes clearly stand out, but also the Pacific Ocean and several European seas are areas of concern.

### 3.5 Shipping noise emissions by vessel type

**Fig. 7:**
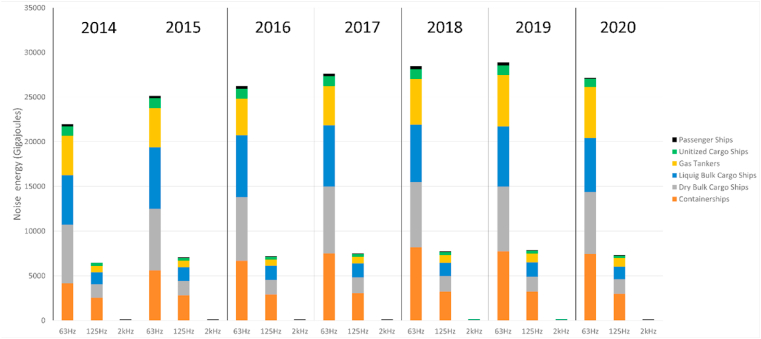
**Global contribution of noise emissions of various ship types 2014–2019 presented as Gigajoules/year**. The aggregated vessel types include the following contributions: Passenger ships include cruise vessels and RoRo/Passenger vessels; unitized Cargo includes the contributions of general cargo ships, RoRo, reefer vessels and vehicle carriers; Liquid tankers include all tanker types carrying liquid cargoes, Gas tankers are given separately in this chart.

One of the advantages of the chosen modeling approach is that it allows determination of noise source energy emissions at ship level as a function of vessel speed, considering the technical characteristics of each vessel in the global fleet. Fig. 7 and Table S2 present examples of this analysis. The height of the 3 bars (Fig. 7) for each year correspond to noise source energy emitted at 63, 125 and 2000 Hz ⅓ octave bands. The noise source energy emissions at 2000 Hz frequency band are significantly lower than for the two other studied bands, because the difference of source level at high frequency can be as much as 30 dB. From Fig. 7 it can be determined that the largest contributions to vessel noise emissions come from container ships and bulk dry cargo carriers, albeit the share of general cargo ships and chemical tankers have increased strongly during the last three years. The large contribution of containerships to overall noise emissions is consistent with earlier findings (Veirs et al., 2018). According to our results, in the list of top 100 noisiest vessels, considering the noise source energy emitted over a period of one year, containerships occupy the first 50 places and represent 80% of the entries on this list. Containerships, gas and crude oil tankers together make 856 entries in the top 1000 noisiest ships.

The increased noise emissions from containerships may be partly because of the increased number of vessels (2014–2019: +12%) or their increased average size, but noise also depends on operating speed. Over 90% of the bulk carriers, gas tankers and vehicle carriers operate with speeds above their estimated cavitation inception speed (CIS), which will lead to large underwater noise emissions. Almost all vessels have decreased their sailing speed during the pandemic, except for LNG tankers. About 95–97% of LNG tanker fleet operated above the estimated CIS, which is in contrast with all other vessel types. Large change in operating speeds were observed for RoRo cargo vessels, of which 75% operated above CIS before the pandemic and only 57% during the COVID-19 pandemic. In case of passenger cruise vessels, the total time spent cruising decreased by 58%, which illustrates the large change in cruise sector operation during 2020. The travel restrictions resulted in a 50% increase in time spent standing still, which reduced the noise emissions from cruise vessels by 73% globally. Contributions of various types of vessels to global shipping noise are reported in Supplementary Material Table S2.

### 3.6 Uncertainties

One of the largest sources of uncertainty in our modeling approach is the determination of cavitation inception speed. This key parameter cannot be obtained from currently available shipping registries, and it is not routinely reported in vessel technical databases. Our previous work (Jalkanen et al., 2018; Karasalo et al., 2017) investigated the performance of our approach in relation to observed noise signatures of ships in the Baltic Sea area and reported largest differences in cases of vessels which use controllable pitch propellers. These vessels do not regulate their velocity by changing propeller rotation speed but change the blade pitch angle instead (Traverso et al., 2015). Since most of the world fleet is equipped with fixed pitch propellers, and the Wittekind model was developed for this kind of vessels, the significance of this uncertainty is likely to be limited. In our previous study (Jalkanen et al., 2018), the sensitivity of noise prediction was tested by changing the cavitation inception threshold speeds by one knot (from 9 to 14 to 10–15 knots) which decreased the noise levels of slow moving cargo ships since more vessel were predicted to operate below the cavitation inception speed. At inventory level, this change reduced the noise emissions at 63 Hz 1/3 octave band by 26%, most notably in vessel classes which have low design speed (crude oil tankers, bulk cargo vessels) (Jalkanen et al., 2018). However, this is unlikely to change the noise trends or the conclusions of this work because similar contributions would be observed for each year. Regardless, it is important to acknowledge the uncertainty concerning the cavitation inception speed which could not be confirmed with measurements in the current work.

Another, yet a smaller source of uncertainty arises from incomplete AIS coverage, gaps in temporal or geographical coverage may occur and these need to be addressed. The impact of temporal gaps in ship activity data are likely to be small, because global AIS service availability was over 99.5 and 99.7 percent for 2019 and 2020, respectively. The model is also capable of solving shortest path – navigation tasks in case of sparse data, avoiding land masses in between the two known vessel positions. Incomplete technical description for vessels is also a source of uncertainty, especially considering the parameters for engine mass. However, the model can estimate missing attributes based on the data from the most similar vessel. Further details are available in our earlier work (Johansson et al., 2017).

It should also be pointed out that not all waterborne traffic uses AIS equipment. The noise emission contribution from military vessels, small fishing boats and recreational craft cannot be fully described with modeling using AIS data as vessel activity description. The results of this paper are likely to underestimate the total waterborne traffic contribution to noise emissions. However, these vessel classes are not directly regulated by the environmental rules of International Maritime Organization conventions which concentrate on shipping.

The uncertainty involved in predicting noise energies is impacted by the model performance. Each of the predicted annual noise source energy totals is subject to uncertainties mentioned above. However, the error involved in prediction of the overall noise trend is less uncertain than that of individual points, if we assume that individual predictions are equally uncertain each year.

## 4 Conclusions

A major result of this modeling study is the quantified increase of underwater noise emissions from shipping and its regional heterogeneity. At the current rate, the global shipping noise emissions double every 11.5 years. The COVID-19 pandemic has temporarily disrupted this increasing trend, but it is expected that noise emissions will increase again once the world economy recovers. In this paper, a rapid increase of shipping noise emissions in near pristine areas, like the Arctic was found, but starting from a low level. Mining operations, oil/gas extraction and vessel routing through Arctic areas will lead to increased shipping noise in these regions.

Out of the 44 studied areas, nine had decreasing shipping noise emissions trend. Further, twenty sea regions were found where doubling of shipping noise emissions takes two decades or longer, whereas 15 remaining sea areas indicate faster increase than that. Of these 15, some European sea areas and especially the Arctic areas were found to have rapid increase of shipping noise emissions during the study period.

Unfortunately, the 15 major sea areas of rapidly increasing noise emissions cover most of the Arctic Ocean, especially if vessel traffic through the Northern Sea Route increases, because the shipping noise emissions in this pristine area have been relatively low. The global pandemic has temporarily reduced the underwater noise emissions back to 2017 levels. The predicted noise source energy emissions in 2020 were reduced by six percent compared to 2019 total at 63 Hz frequency band. Largest changes were predicted for passenger cruisers, vehicle carriers and ropax vessels.

According to the model, the contribution of container ships, bulk cargo ships and tankers to underwater noise emissions is over 68% when the results are aggregated by vessel category. Based on the contribution of individual vessels, the 50 largest noise source energy emitters are all containerships. This metric considers both the source level (dB), and the time integration of noise emissions over the period of one year. It cannot be interpreted that the containerships have the highest source levels, because both the source level and active time contribute to total noise source energy emitted. Regardless, vessel design, technical and operational measures are necessary to avoid rapid increase of shipping noise emissions which were already observed before the pandemic.

The increasing shipping noise emissions are highly variable in different sea regions.

## Funding

This project has received funding from the 10.13039/100010661European Union's Horizon 2020 Research And Innovation Programme under grant agreement # 764553 (AIRCOAT project). This work reflects only the authors' view and INEA is not responsible for any use that may be made of the information it contains. The authors thank the Working Group SHIP of the International Council for the Exploration of the Sea (ICES) for facilitating this research. We thank the anonymous referees for their valuable feedback.

## Author contributions

J-PJ was responsible for designing the study, global noise modeling and overall responsibility of the work. EM and LJ data processing and STEAM model updates. MA and PS methodology development and result analysis. All authors have contributed to the manuscript writing process.

## Declaration of competing interest

The authors declare that they have no known competing financial interests or personal relationships that could have appeared to influence the work reported in this paper.

## Data Availability

The data associated to this article can be found with Zenodo, https://doi.org/10.5281/zenodo.6513401. Model source code and input datasets are subject to contracts with third parties and cannot be shared.
