# Supplementary material for: Underwater noise emissions from ships during 2014–2020
Source: Environ Pollut. 2022 Oct 15;311:119766. doi: 10.1016/j.envpol.2022.119766 (PMC9489924; doi:10.1016/j.envpol.2022.119766)
Supplement: Multimedia Component 2 [file mmc2.docx]

**Supplement**

**A short summary of STEAM modeling approach concerning underwater noise**

An implementation of the Wittekind noise source model in STEAM is described in our earlier work(Jalkanen et al., 2018), but a short summary is provided in this Supplement. STEAM uses AIS data from terrestrial and satellite receivers. Access to these data were bought from Orbcomm Ltd. In addition, STEAM uses IHS Markit database of ships to describe the technical features of each vessel in the world fleet. An annual license is required to use IHS Markit data.

In AIS data, two options for vessel identification are available, namely IMO (International Maritime Organization) registry number and MMSI (Maritime Mobile Service Identity), which can be used as search keys when accessing vessel technical data. In STEAM, the primary search key is the IMO number, which remains constant from vessel construction to vessel scrapping. The secondary search key is the MMSI code, which will change when a vessel changes its flag state. Emissions of vessels using more than one MMSI code, but the same IMO number are summed together. If a vessel cannot be reliably identified, then it is assumed to be a small emission source.

Equations from the Wittekind (Wittekind, 2014) describing the source levels (in dB re 1 µPa) require that the vessel block coefficient, displacement, engine mounting type, number of operating engines, engine masses and cavitation inception speed are available. In STEAM, block coefficient is estimated using the method suggested by Watson (Watson, 1998) With this approach, the block coefficient can be estimated from:

$$C_{b}=0.7+\frac{1}{8}atan\left( \frac{23-100F_{n}}{4} \right)$$

where F_n_ is the Froude number. Vessel displacement is evaluated with

$$\nabla=Length*Breadth*Draught*C_{b}$$

Engine mounting type is determined from engine specifications using the classification mentioned in Rowen (Rowen, 2003) and Kuiken (Kuiken K., 2008). In short, rigid mounting is assumed for large 2-stroke engines, and resilient mounting for medium- and high-speed diesel engines.

Engine mass is determined from engine catalogue (Barnes et al., 2005) and engine manufacturer documentation. This allows for the determination of main engine mass for approximately two thirds of the world fleet, and the rest are determined with mass/power relationships based on these known entries. More details, and the associated equations can be found in our earlier work (Jalkanen et al., 2018).

In addition to main engine mass, also the number of operating engines is required. This is determined during a regular STEAM run from the required thrust, considering propeller and transmission losses when evaluating the required propulsion power to propel the vessel at speed indicated in AIS data, and considering the machinery details of the ship. In the case of multiple main engines, the minimum number of engines is assumed to be in use(Jalkanen et al., 2012). Engine loads of operational engines are allowed to change dynamically up to 0.85, which is a threshold value for making additional engines operational. Regardless, the hard limit for engine power is the installed engine power determined from engine specifications. A limitation of this approach is that all main engines are considered equal, whereas in reality engine setup may include combinations of different sizes of main engines.

For cavitation inception speed (V_CIS_) estimate, a connection between cavitation inception and vessel block coefficient was developed based on discussions with a propulsion system manufacturer(Jalkanen et al., 2018). The relation used in our earlier study, is:

$$V_{CIS}=min\left\{ max\left[ \left( 1.42-1.2c_{b} \right)*V_{d};9 \right];14 \right\}$$

For estimates of V_CIS_, block coefficient and vessel design speed (V_d_) are required. The design speed is obtained from IHS Markit data. It should be noted that with this approach, all vessels cavitate when traveling over 14 knots, but for slow cargo vessels V_CIS_ may be closer to nine knots. A comparison of predicted noise source levels to hydrophone measurements was reported by Karasalo(Karasalo et al., 2017).

The Wittekind source model considers three contributions to underwater noise, which are the low and high frequency cavitation and machinery. For each vessel, the source level is estimated using his equations (Wittekind, 2014)and these are converted to noise source energy using:

$${SL}_{k}\left[ dB re 1m, 1\mu Pa \right]=10{log}_{10}\frac{P_{k}}{P_{ref}}$$

where $P_{ref}=\frac{4\pi p_{ref}^{2}}{\rho c}$ is the reference power, ρ and c are density and speed of sound, while p_ref_ is equal to 1 microPa. We assume that all noise sources are uncorrelated, and the total emitted power from all M ships in area A at time t is given by:

$$P_{k}^{tot}\left( t \right)=\sum_{m=1}^{M} P_{k,m}(t)$$

where P_k,m_(t) is the sound power (in J s^-1^) emitted by ship m. With these definitions, noise energies can be drawn as a map, which is a visual aid to quickly assess the areas of high noise emissions and spot noise trends as a function of time. It should be noted that source levels (in dB) as a function of time and location can be provided as output to noise propagation modeling, but this output option was not used in the current study because propagation studies were not made.
